# Positive effects of student-led health promotion activities at mass gathering events on health modification behaviors

**DOI:** 10.1093/pubmed/fdaf159

**Published:** 2025-12-23

**Authors:** Michelle Anne Stubbs, Mathieu Figeys, Natalie Russell-Hurst, Lee Lethbridge, Cassie Taylor, Bethany Porteous, Alison Hutton

**Affiliations:** University of Newcastle, College of Health, Medicine and Wellbeing, School of Nursing and Midwifery, University Drive, Callaghan NSW 2308, Australia; Hunter Medical Research Institute, Lookout Road, New Lambton Heights NSW 2305, Australia; University of Newcastle, College of Health, Medicine and Wellbeing, School of Nursing and Midwifery, University Drive, Callaghan NSW 2308, Australia; Hunter Medical Research Institute, Lookout Road, New Lambton Heights NSW 2305, Australia; Central Coast Research Institute, 77A Holden Street, Gosford NSW 2250, Australia; University of Newcastle, College of Health, Medicine and Wellbeing, School of Nursing and Midwifery, University Drive, Callaghan NSW 2308, Australia; University of Newcastle, College of Health, Medicine and Wellbeing, School of Nursing and Midwifery, University Drive, Callaghan NSW 2308, Australia; University of Newcastle, College of Health, Medicine and Wellbeing, School of Nursing and Midwifery, University Drive, Callaghan NSW 2308, Australia; University of Newcastle, College of Health, Medicine and Wellbeing, School of Nursing and Midwifery, University Drive, Callaghan NSW 2308, Australia; University of Newcastle, College of Health, Medicine and Wellbeing, School of Nursing and Midwifery, University Drive, Callaghan NSW 2308, Australia; Western Sydney University, Faculty of Health, School of Nursing and Midwifery, 255 Elizabeth Street, Sydney 2000, Australia

**Keywords:** health modification behaviors, health promotion, mass gathering events, structural equation model, student-led health initiative

## Abstract

The ‘Pit Stop for Health’ initiative is a student-led health promotion program providing opportunistic health screenings and behavioral counseling at mass gathering events. This cross-sectional observational study surveyed 369 participants across agricultural fairs, sporting events, and university activities. Structural equation modeling identified multiple pathways influencing satisfaction, health modification, and health behavior’s, with the overall model explaining 58.8% of the variance (*R*^2^ = 0.588). Satisfaction with the ‘Pit Stop for Health’ initiative significantly predicted health modification (β = 0.157, SE = 0.068, CR = 2.316, *P* = .021) and the likelihood of recommending the activity to others (β = 0.812, SE = 0.138, CR = 5.877, *P* < .001), while follow-up behaviors were strongly associated with future health modification (β = 0.854, SE = 0.116, CR = 7.380, *P* < .001) and seeking medical advice (β = 0.967, SE = 0.107, CR = 9.050, *P* < .001). The model demonstrated excellent fit (χ^2^ = 33.736, df = 24, *P* = .090), with age negatively correlated with follow-up behaviors (r = −0.137, *P* = .028), and no significant associations found for gender or self-reported health importance. Findings highlight the public health value of student-led, reciprocal service-learning models in delivering preventive interventions in non-clinical community settings. Such initiatives not only strengthen public engagement with health services but also provide meaningful experiential learning for future health professionals. By addressing preventive health needs in accessible, resource-constrained environments, ‘Pit Stop for Health’ contributes to population-level health promotion. Further research examining cultural and ethnic influences is warranted to enhance the inclusivity, relevance, and scalability of similar interventions in diverse communities.

**Contribution to Health Promotion**
 

- Promoted health behavior changes by offering opportunistic health screenings and lifestyle modification counseling at mass gathering events.
- Increased access to preventive healthcare services, especially in resource-limited settings, through a cost-effective student-led initiative.
- Fostered community engagement in health promotion while simultaneously enriching nursing students’ educational experiences.
- Enhanced public awareness of health importance through innovative, context-sensitive interventions at diverse public events.
- Supported healthcare systems by addressing preventive health needs and improving follow-up behaviors among participants.

## Background

Opportunistic health assessments, which involve the provision of health checks in non-clinical settings without prior appointment, represent an important strategy for increasing early detection and preventive health engagement.^1^^,^^2^ These assessments, when delivered in accessible and informal environments, can serve as a ‘teachable moment,’ prompting individuals to reflect on their current health status and consider behavior change.^3^^,^^4^ A teachable moment during an opportunistic health check refers to a naturally occurring situation—often triggered by a health concern or unexpected finding—where an individual becomes more receptive to learning about their health and motivated to adopt healthier behaviors.^5^^,^^6^

Mass gathering events (MGEs)—such as music festivals, community fairs, and sporting events—are increasingly recognized as promising venues for such interventions.^7^^,^^8^ MGEs draw diverse populations and offer a relaxed, socially engaging environment that can reduce perceived barriers to care, including fear, stigma, or mistrust of formal healthcare systems.^9^^,^^10^ Recent studies have highlighted the potential of MGE-based health interventions to influence health-seeking behavior.^11^^,^^12^ These interventions may be particularly impactful for populations less likely to engage with traditional healthcare services, such as young adults and individuals from socioeconomically disadvantaged backgrounds.^13^ Moreover, they offer a valuable experiential learning opportunity for healthcare students, who develop critical skills in health communication and preventive care delivery.^14^

Opportunistic health assessments may also be led by students. Student-led health promotion activities provide a sustainable and cost-effective way to deliver focused public health messages and community engagement. Student-led activities may be implemented through multiple initiatives such as community health clinics and visits, large-scale events, and through the use of various digital technologies.^15–17^ Local communities are considered a place for learning in real-world contexts, where students, nursing educators, community members, and people from other sectors all participate in the learning experience.^18^^,^^19^ Community benefits of student-led activities include increased access to health promotion and disease prevention services within regional contexts.^20^

Despite increasing interest in the use of MGEs as platforms for health promotion, there remains a notable gap in literature specifically examining the influence of opportunistic health assessments at these events on subsequent health-seeking behavior. While few studies have documented the feasibility of delivering preventive health interventions at music festivals, agricultural shows, and sporting events, few have systematically evaluated their effectiveness in motivating individuals to engage with formal healthcare services following the event.^9^^,^^10^ One significant research gap that remains is the limitation their effectiveness and scalability, and the lack of standardized measurement tools to assess behavioral outcomes following health interventions delivered in these settings.^21^ Most studies rely on self-reported data without longitudinal follow-up, making it difficult to determine whether health behavior changes are sustained over time.^22^ Additionally, population targeting strategies are underdeveloped; while MGEs attract diverse attendees, few studies have explored how demographic factors such as age, gender identity, cultural background, or health literacy influence engagement with health checks. For example, Haută *et al*.^6^ identified age and gender disparities in healthcare needs at a religious MGE, but did not examine how these differences affect receptiveness to health education or follow-up behaviors 1. There is also limited exploration of teachable moments—those emotionally salient or contextually relevant opportunities for health promotion—which are often present but not systematically leveraged during MGEs. Furthermore, the integration of behavioral science frameworks such as the Health Belief Model (HBM) or COM-B remains rare, leaving a theoretical gap in understanding how and why individuals respond to opportunistic health interventions in these dynamic environments. Most existing research has focused on the operational logistics or immediate outcomes of mass gathering health services, such as injury management and harm minimization,^23^ rather than on long-term behavioral change. Furthermore, little is known about how different population groups—particularly younger adults and under-screened demographics—perceive and respond to health information delivered in these non-traditional settings.

As a result, there is limited evidence to guide best practice in designing opportunistic health interventions at MGEs that translate into sustained engagement with preventive care. Addressing this gap is important for harnessing the full potential of mass gatherings as public health outreach platforms and advancing health equity through early intervention. Thus, this study aimed to investigate the influence of satisfaction from student-led opportunistic health assessments at MGEs in promoting health and influencing health behavior through a secondary analysis. This study further aimed to evaluate the relationship between participants’ satisfaction with the ‘Pit Stop for Health’ initiative at MGEs and their engagement in subsequent health-related behavioral changes. Secondary objectives included examining associations between satisfaction and future service use, the mediating role of follow-up behaviors, and the influence of demographic factors and self-reported health importance on outcomes. We hypothesized that satisfaction in ‘Pit Stop for Health’ increased health-related behavioral changes.

## Methods

### Study design

Between March and September 2024, an analytical cross-sectional study was undertaken. The study was conducted in accordance with the Declaration of Helsinki and reported using the Strengthening the Reporting of Observational Studies in Epidemiology guidelines to ensure methodological rigor. Ethical approval was obtained from the University of Newcastle Human Research Ethics Committee (Approval No. H-2021-0160).

### Participants and recruitment

Recruitment was undertaken via simple random sampling. Participants were eligible to take part if they were aged 18 years or older. Exclusion criteria included individuals under 18 years of age, non-English speakers, those with cognitive impairments affecting consent, or individuals experiencing acute medical issues. Consent was implied via the completion of the ‘Pit Stop for Health’ activity. At all MGEs, ‘Pit Stop for Health’ activity stations were strategically placed in central locations, as designated by the event organizers. These stations were visible from multiple vantage points due to prominent signage specifically identifying the ‘Pit Stop for Health’ student-led activity. MGEs included a three-day regional agricultural and horticultural event, a motor car sporting event, two non-consecutive university promotional and information events, and a two-day surfing festival.

### Data collection

Using a checklist (see Supplementary A) as a conversation starter, students assess sleep, social supports (someone to talk to), blood pressure, waist circumference, alcohol consumption and exercise. Students engage the public in discussions about health focusing on lifestyle modifications and early detection strategies. ‘Pit Stop for Health’ is a reciprocal service-learning model that supports pressured healthcare systems and compliments limited professional experience placement hours of pre-registration nursing programs.^24–26^ Electronic data was gathered using Survey Monkey after receiving Pit Stop for Health services from student volunteers. The study used a 14-item, 5-point Likert scale questionnaire (1 = Extremely unlikely, 2 = Somewhat unlikely, 3 = Neutral, 4 = Somewhat likely, and 5 = Extremely likely). Categorized Likert scale questions include: (i) are you likely to follow up with your doctor or health professional after visiting us? (ii) would you consider having an annual health check after visiting the us? (iii) are you likely to seek any other form of medical advice or assistance? and (iv) would you visit the ‘Pit Stop for Health’ again? to evaluate the intentions of participants to seek healthcare or visit a medical practitioner after participating in the health promotion activity. In addition, attitudinal questions were grouped together to assess the attitude of participants towards their health via the questions: (v) would you recommend ‘Pit Stop for Health’ to your friends/family? (vi) would you modify your health behaviors in any way? and (vii) how important is your health to you?

Several strategies were employed to minimize potential sources of bias during data collection, ensuring the validity and reliability of the results. The survey design emphasized the use of clear, neutral language to avoid leading questions, with a logically structured format to reduce priming effects. Comprehensive response options, including choices like ‘Not Applicable’ or ‘Prefer Not to Answer,’ were provided to accommodate diverse responses. Sensitive topics were addressed through carefully framed questions that normalized a range of answers, and immediate completion of the survey was employed to reduce recall bias. Pre-testing was conducted to identify and revise any unclear or biased items. Opportunistic sampling^27^ was used to ensure representative participation, and the sample size was sufficiently large to enhance reliability.

Data collection adhered to a standardized protocol with neutral instructions to maintain consistency, while anonymity was ensured to mitigate social desirability bias. Surveys were administered at appropriate times to minimize external influences, with cultural and temporal sensitivities considered. Students completed a 1-hour training session focused on non-priming techniques, confidentiality, cultural sensitivity, and consistent communication. Training included role-plays and standardized scripts to support consistent question delivery. Students asked questions using a neutral tone and followed the approved screening tool verbatim to minimize bias. These comprehensive efforts effectively reduced bias and upheld the integrity of the research.

### Data analysis

Descriptive statistics, including measures of central tendency, normality, and frequencies reported in the questionnaires, as well as the amount of missing data, were calculated in SPSS Version 30 (IBM Corp., Armonk, NY). Measures of central tendency are reported as frequency counts with corresponding percentages, medians, means, standard deviations, and ranges. Missing data was addressed through Full-information maximum likelihood estimation to retain cases with missing data (~1% of the dataset). A response rate was not calculated due to voluntary participation without a defined sampling frame.

To determine the patterns and relationships between and among variables, structural equation modeling was conducted in IBM Amos (Version 30, Armonk, NY), using the dataset imputed for missing values. Variables related to Health Importance (Self-reported health importance, number of medical checks annually), Pit Stop Satisfaction (Pit Stop recommendation, Would Visit Pit Stop again), and Follow-up (Physician follow up after Pit Stop, follow-up with other medical/allied health professionals) were used to predict patterns of health modification (including self-reported health modification, considering annual health check-ups), while controlling for age and gender in the model.

Age and gender were utilized as covariance parameters, thus without a direction of causation implied. Observed variables included median scores on Likert scale questions. The latent factor of health modification included the Likert questions of health modification and consider annual check-ups; The latent factor of Follow-Up included median reported willingness to follow-up with a physician as well as seek medical advice; Health importance as a latent variable included the Likert components of median self-reported health importance as well as number of health checks per year; Lastly, the latent variable of Pit Stop for Health included Likert scale median ranks of visiting Pit Stop for Health again, and recommending Pit Stop for Health to others.

Using a 25-iteration limit, maximum likelihood regression beta coefficients from the structural equation model were obtained, in addition to model effects, and metrics of model fit. The criterion proposed by Hu and Bentler (1999) were utilized to establish model fit. Significance was determined at *P* < .05 (Hu and Bentler, 1999).

### Sample size

A total of 369 responses were recorded in the initial study. A sample size of 200 or more, or a minimum of 10 individuals per parameter, is considered a general rule for structural equating models to maintain power and minimize estimate bias;^28^^,^^29^ therefore, the sample size of 369 is appropriate for the exploratory structural equating model.^30^

## Results

### Demographics

A total of 369 responses of individuals who received community health screening and education through Pit Stop for Health were analysed. Data was determined to be normally distributed visually, with an absolute skew value less than 2, determining normality.^31^^,^^32^ Demographic characteristics are reported below in Table 1. The majority of responses were from females within a younger adult age demographic, with a notable decline in participation observed among older adults. Table 2 describes the central tendency of the questionnaire variables, alongside the total of the administered Likert scale, with the frequency of reported missing data.

**Table 1:** Note—Data presented as frequency counts with corresponding percentages. Male and female refer to individuals who identify with the gender typically associated with their biological sex. Transgender refers to individuals whose gender identity differs from the sex they were assigned at birth. Non-binary describes people who do not identify exclusively as male or female. Cisgender refers to individuals whose gender identity matches the sex they were assigned at birth (association, 2021^33^^,^^34^).

Category	*N*	%
Age Group		
18–25	127	34.4%
26–35	59	16.0%
36–45	41	11.1%
46–55	64	17.3%
56–65	40	10.8%
66–75	32	8.7%
76–85	6	1.6%
Gender (Self-reported)		
Male	140	37.9%
Female	222	60.2%
Non-binary	4	1.1%
Transgender	2	0.5%
Cisgender	1	0.3%

**Table 2:** Note—Data presented as median, minimum, maximum, and sample size, stratified by self-reported gender and age.

Pit Stop for Health Responses (Medians)										
Gender (Self-reported)	Age		Health Importance	Number health checks per year	Health modification	Doctor Followup	Consider AnnualCheck	Seek MedicalAdvice	Visit Pitstop Again	Recommend Pitstop
Male	18–25	Median	4.00	2.00	3.00	3.00	2.00	3.00	4.00	4.00
		Minimum	1.00	1.00	1.00	1.00	1.00	1.00	2.00	2.00
		Maximum	5.00	11.00	5.00	5.00	5.00	5.00	5.00	5.00
		N	44.00	45.00	45.00	46.00	45.00	46.00	46.00	46.00
	26–35	Median	4.00	1.00	3.00	3.00	2.00	2.50	4.00	4.00
		Minimum	1.00	1.00	1.00	1.00	1.00	1.00	2.00	2.00
		Maximum	5.00	10.00	5.00	5.00	5.00	5.00	5.00	5.00
		N	21.00	20.00	21.00	21.00	22.00	22.00	22.00	21.00
	36–45	Median	3.00	2.00	4.00	3.00	1.50	2.00	5.00	5.00
		Minimum	1.00	1.00	2.00	1.00	1.00	1.00	1.00	1.00
		Maximum	5.00	6.00	5.00	5.00	5.00	5.00	5.00	5.00
		N	16.00	16.00	16.00	16.00	16.00	16.00	15.00	16.00
	46–55	Median	5.00	1.50	4.00	3.00	2.00	2.00	5.00	5.00
		Minimum	1.00	1.00	1.00	1.00	1.00	1.00	1.00	1.00
		Maximum	5.00	8.00	5.00	5.00	5.00	5.00	5.00	5.00
		N	26.00	26.00	25.00	25.00	25.00	25.00	25.00	25.00
	56–65	Median	5.00	1.50	4.00	2.00	1.00	2.00	5.00	5.00
		Minimum	1.00	1.00	1.00	1.00	1.00	1.00	1.00	1.00
		Maximum	5.00	10.00	5.00	5.00	5.00	5.00	5.00	5.00
		N	17.00	16.00	16.00	17.00	17.00	16.00	17.00	17.00
	66–75	Median	5.00	1.50	3.00	2.00	1.00	2.00	5.00	5.00
		Minimum	1.00	1.00	2.00	1.00	1.00	1.00	1.00	1.00
		Maximum	5.00	3.00	5.00	5.00	5.00	5.00	5.00	5.00
		N	11.00	10.00	11.00	11.00	11.00	11.00	11.00	11.00
	76–85	Median	4.50	4.00	4.00	2.00	1.50	2.00	4.50	4.50
		Minimum	4.00	2.00	4.00	1.00	1.00	1.00	4.00	4.00
		Maximum	5.00	6.00	4.00	3.00	2.00	3.00	5.00	5.00
		N	2.00	2.00	1.00	2.00	2.00	2.00	2.00	2.00
	Total	Median	4.00	2.00	4.00	3.00	2.00	3.00	5.00	5.00
		Minimum	1.00	1.00	1.00	1.00	1.00	1.00	1.00	1.00
		Maximum	5.00	11.00	5.00	5.00	5.00	5.00	5.00	5.00
		N	137.00	135.00	135.00	138.00	138.00	138.00	138.00	138.00
Female	18–25	Median	4.00	2.00	4.00	3.00	2.00	3.00	5.00	5.00
		Minimum	1.00	1.00	1.00	1.00	1.00	1.00	1.00	1.00
		Maximum	5.00	11.00	5.00	5.00	5.00	5.00	5.00	5.00
		N	76.00	75.00	76.00	76.00	76.00	76.00	76.00	75.00
	26–35	Median	4.00	3.00	4.00	3.00	2.00	2.00	5.00	5.00
		Minimum	1.00	1.00	1.00	1.00	1.00	1.00	1.00	1.00
		Maximum	5.00	11.00	5.00	5.00	5.00	5.00	5.00	5.00
		N	36.00	35.00	36.00	36.00	36.00	36.00	36.00	36.00
	36–45	Median	5.00	2.00	4.00	3.00	2.00	3.00	4.00	5.00
		Minimum	1.00	1.00	1.00	1.00	1.00	2.00	1.00	1.00
		Maximum	5.00	6.00	5.00	5.00	5.00	5.00	5.00	5.00
		N	25.00	25.00	25.00	25.00	25.00	25.00	25.00	25.00
	46–55	Median	4.50	2.00	4.00	3.00	2.00	3.00	5.00	5.00
		Minimum	1.00	1.00	1.00	1.00	1.00	1.00	1.00	1.00
		Maximum	5.00	11.00	5.00	5.00	5.00	5.00	5.00	5.00
		N	38.00	37.00	38.00	38.00	38.00	38.00	37.00	38.00
	56–65	Median	5.00	1.00	4.00	2.50	2.00	2.00	4.00	5.00
		Minimum	1.00	1.00	1.00	1.00	1.00	1.00	1.00	1.00
		Maximum	5.00	6.00	5.00	5.00	5.00	5.00	5.00	5.00
		N	22.00	22.00	22.00	22.00	22.00	21.00	22.00	22.00
	66–75	Median	5.00	2.00	3.00	2.00	1.00	2.50	5.00	5.00
		Minimum	1.00	1.00	1.00	1.00	1.00	1.00	1.00	1.00
		Maximum	5.00	11.00	5.00	5.00	5.00	5.00	5.00	5.00
		N	21.00	21.00	20.00	21.00	21.00	20.00	21.00	21.00
	76–85	Median	4.50	3.00	4.00	3.50	1.00	3.00	5.00	5.00
		Minimum	3.00	2.00	2.00	1.00	1.00	1.00	4.00	5.00
		Maximum	5.00	5.00	5.00	5.00	5.00	5.00	5.00	5.00
		N	4.00	4.00	4.00	4.00	4.00	4.00	4.00	4.00
	Total	Median	5.00	2.00	4.00	3.00	2.00	3.00	5.00	5.00
		Minimum	1.00	1.00	1.00	1.00	1.00	1.00	1.00	1.00
		Maximum	5.00	11.00	5.00	5.00	5.00	5.00	5.00	5.00
		N	222.00	219.00	221.00	222.00	222.00	220.00	221.00	221.00
Non-binary	18–25	Median	4.00	1.00	3.00	3.00	3.00	4.00	5.00	5.00
		Minimum	3.00	1.00	3.00	3.00	1.00	3.00	3.00	4.00
		Maximum	5.00	5.00	4.00	5.00	5.00	5.00	5.00	5.00
		N	3.00	3.00	3.00	3.00	3.00	2.00	3.00	3.00
	56–65	Median	5.00	2.00	5.00	5.00	1.00	1.00	5.00	5.00
		Minimum	5.00	2.00	5.00	5.00	1.00	1.00	5.00	5.00
		Maximum	5.00	2.00	5.00	5.00	1.00	1.00	5.00	5.00
		N	1.00	1.00	1.00	1.00	1.00	1.00	1.00	1.00
	Total	Median	4.50	1.50	3.50	4.00	2.00	3.00	5.00	5.00
		Minimum	3.00	1.00	3.00	3.00	1.00	1.00	3.00	4.00
		Maximum	5.00	5.00	5.00	5.00	5.00	5.00	5.00	5.00
		N	4.00	4.00	4.00	4.00	4.00	3.00	4.00	4.00
Trans-gender	18–25	Median	3.50	3.50	3.50	2.00	2.00	2.00	4.50	4.50
		Minimum	3.00	3.00	3.00	2.00	2.00	2.00	4.00	4.00
		Maximum	4.00	4.00	4.00	2.00	2.00	2.00	5.00	5.00
		N	2.00	2.00	2.00	1.00	1.00	2.00	2.00	2.00
	Total	Median	3.50	3.50	3.50	2.00	2.00	2.00	4.50	4.50
		Minimum	3.00	3.00	3.00	2.00	2.00	2.00	4.00	4.00
		Maximum	4.00	4.00	4.00	2.00	2.00	2.00	5.00	5.00
		N	2.00	2.00	2.00	1.00	1.00	2.00	2.00	2.00
Cis-gender	26–35	Median	4.00	2.00	NR	4.00	3.00	2.00	4.00	4.00
		Minimum	4.00	2.00	NR	4.00	3.00	2.00	4.00	4.00
		Maximum	4.00	2.00	NR	4.00	3.00	2.00	4.00	4.00
		N	1.00	1.00	NR	1.00	1.00	1.00	1.00	1.00
	Total	Median	4.00	2.00	NR	4.00	3.00	2.00	4.00	4.00
		Minimum	4.00	2.00	NR	4.00	3.00	2.00	4.00	4.00
		Maximum	4.00	2.00	NR	4.00	3.00	2.00	4.00	4.00
		N	1.00	1.00	NR	1.00	1.00	1.00	1.00	1.00
Total	18–25	Median	4.00	2.00	4.00	3.00	2.00	3.00	5.00	5.00
		Minimum	1.00	1.00	1.00	1.00	1.00	1.00	1.00	1.00
		Maximum	5.00	11.00	5.00	5.00	5.00	5.00	5.00	5.00
		N	125.00	125.00	126.00	126.00	125.00	126.00	127.00	126.00
	26–35	Median	4.00	2.00	4.00	3.00	2.00	2.00	4.00	5.00
		Minimum	1.00	1.00	1.00	1.00	1.00	1.00	1.00	1.00
		Maximum	5.00	11.00	5.00	5.00	5.00	5.00	5.00	5.00
		N	58.00	56.00	57.00	58.00	59.00	59.00	59.00	58.00
	36–45	Median	4.00	2.00	4.00	3.00	2.00	3.00	4.00	5.00
		Minimum	1.00	1.00	1.00	1.00	1.00	1.00	1.00	1.00
		Maximum	5.00	6.00	5.00	5.00	5.00	5.00	5.00	5.00
		N	41.00	41.00	41.00	41.00	41.00	41.00	40.00	41.00
	46–55	Median	5.00	2.00	4.00	3.00	2.00	3.00	5.00	5.00
		Minimum	1.00	1.00	1.00	1.00	1.00	1.00	1.00	1.00
		Maximum	5.00	11.00	5.00	5.00	5.00	5.00	5.00	5.00
		N	64.00	63.00	63.00	63.00	63.00	63.00	62.00	63.00
	56–65	Median	5.00	1.00	4.00	2.50	2.00	2.00	5.00	5.00
		Minimum	1.00	1.00	1.00	1.00	1.00	1.00	1.00	1.00
		Maximum	5.00	10.00	5.00	5.00	5.00	5.00	5.00	5.00
		N	40.00	39.00	39.00	40.00	40.00	38.00	40.00	40.00
	66–75	Median	5.00	2.00	3.00	2.00	1.00	2.00	5.00	5.00
		Minimum	1.00	1.00	1.00	1.00	1.00	1.00	1.00	1.00
		Maximum	5.00	11.00	5.00	5.00	5.00	5.00	5.00	5.00
		N	32.00	31.00	31.00	32.00	32.00	31.00	32.00	32.00
	76–85	Median	4.50	3.00	4.00	3.00	1.00	2.50	5.00	5.00
		Minimum	3.00	2.00	2.00	1.00	1.00	1.00	4.00	4.00
		Maximum	5.00	6.00	5.00	5.00	5.00	5.00	5.00	5.00
		N	6.00	6.00	5.00	6.00	6.00	6.00	6.00	6.00
	Total	Median	4.00	2.00	4.00	3.00	2.00	3.00	5.00	5.00
		Minimum	1.00	1.00	1.00	1.00	1.00	1.00	1.00	1.00
		Maximum	5.00	11.00	5.00	5.00	5.00	5.00	5.00	5.00
		N	366.00	361.00	362.00	366.00	366.00	364.00	366.00	366.00

NR, not reported.

### Path analysis

The structural equating modeling examined multiple pathways influencing satisfaction, health modification, and health behaviors. Results demonstrate the relationship between overall satisfaction with ‘Pit Stop for Health’ and Health Modification was significant (β = 0.157, SE = 0.068, CR = 2.316, *P* = .021). Satisfaction with ‘Pit Stop for Health’ increased and was associated with the likelihood of recommending the activity to others (β = 0.812, SE = 0.138, CR = 5.877, *P* < .001).

A significant effect was observed for future follow up and health modification behaviors (β = 0.854, SE = 0.116, CR = 7.380, *P* < .001), however, self-reported level of health importance was not statistically significantly related to health modification. Follow Up was significantly related to seeking medical advice (β = 0.967, SE = 0.107, CR = 9.050, *P* < .001), whereas self-reported level of health showed no association with the number of yearly checkups. An overall *R*^2^ = 0.588 was obtained, with fixed paths removed, allowing a free estimate of relationships of variance explained. Refer to Table 3.

**Table 3:** Note—Overall *R*^2^ = 0.588. Structural paths were specified a priori based on the COM-B model.

Path	Estimate (β)	SE	CR	*P*-value
Pit Stop Satisfaction → Health Modification	0.157	0.068	2.316	0.021
Follow Up → Health Modification	0.854	0.116	7.380	< 0.001
Health Importance→ Health Modification	−0.047	0.265	−0.177	0.860
Pit Stop Satisfaction → Pit Stop Recommendation	0.812	0.138	5.877	< 0.001
Follow Up → Seek Medical Advice	0.967	0.107	9.050	< 0.001
Self-Report of Health → Health Check Year	0.024	0.141	0.170	0.865

### Covariance of age and gender

Age and gender were evaluated as predictors within the model, with relationships reported through correlations. Age had a significant negative correlation with follow-up behaviors (*r* = −.137, *P* = .028). Age was not significantly correlated with satisfaction or other model predictors. In addition, gender was not significantly associated with any variable.

### Model fit

Utilizing multiple goodness of fit indices, the model had an excellent fit to the data. This is supported by a non-significant χ^2^ = 33.736 (*P* = .090; df = 24), in addition to multiple other indices reported in Table 4. Overall, the results report that the model provides a robust representation of data.

**Table 4:** Note: ^1^Suggested recommended values reported in Hosseinabadi and colleagues (2018) and Hu and Bentler (1999).^35^

Fit Index	Value	Recommended Value^1^	Confirm/Reject
Chi-Square (χ^2^)	33.726	–	Confirm (*P* = .090)
Degrees of Freedom (df)	24	–	–
χ^2^/df ratio	1.405	< 3	Confirm
Root mean square error of approximation (RMSEA)	0.033	≤ 0.10	Confirm
Comparative Fit Index (CFI)	0.985	> 0.9	Confirm
Tucker-Lewis Index (TLI)	0.972	> 0.9	Confirm
Goodness of Fit Index (GFI)	0.983	> 0.9	Confirm
Adjusted Goodness of Fit Index (AGFI)	0.961	> 0.9	Confirm
Standardized Root Mean Square Residual (SRMR)	0.0302	≤ 0.08	Confirm

## Discussion

The aim of this study was to investigate the influence of satisfaction of ‘Pit Stop for Health’ on health-related behavioral changes and revisiting the activity. Data from 369 participants who utilized the student-led ‘Pit Stop for Health’ initiative were analysed, with most respondents being between 18–55 years old. A structural equating model with an excellent fit was utilized to explore this.

### Main findings of this study

Satisfaction with ‘Pit Stop for Health’ was a positive predictor towards health modification behaviors. Satisfaction was also associated with the likelihood of referral of others to utilize the service. These results may be explained by the Theory of Planned Behavior (TPB) that posits that behavioral intentions are influenced by attitudes toward the behavior, subjective norms, and perceived behavioral control.^36^ In this study, satisfaction with the ‘Pit Stop for Health’ service likely enhanced participants’ attitudes and perceived control, thereby increasing the likelihood of engaging in health-modifying behaviors and follow-up actions. However, the lack of association between self-reported health importance and behavior change may reflect a disconnect between intention and action, a known limitation in TPB applications.^36^

In addition to the TPB, the HBM may also explain study findings. The HBM suggests that health behaviors are influenced by perceived susceptibility, severity, benefits, and barriers, along with cues to action and self-efficacy.^37^ Satisfaction with the service may have acted as a cue to action, reinforcing perceived benefits and reducing perceived barriers.^37^ However, the absence of a relationship between self-reported health importance and behavior change could indicate that perceived susceptibility or severity was not sufficiently activated during the intervention.

A significant effect was observed for future follow up with healthcare providers and health modification behaviors after attending ‘Pit Stop for Health’. Of interest, self-reported health importance was not related to health modification, or the frequency of self-reported health checkups. Age was found to be negatively correlated with following up with health services, with younger participants being more likely to follow-up with health services after visiting ‘Pit Stop for Health.’

Overall, confirming our hypothesis, satisfaction with ‘Pit Stop for Health’ increased components of health modifying behaviors. The effects of self-reported health modifying behaviors is a dynamic and complex influence between constructs, which was found not to be associated between constructs in this study. More so, gender had no effect in the model. This is a curious finding, as gender is known to be a potential confounder in health modification due to its complex influence on health outcomes through biological and social determinants.^38^ Biologically, gender differences manifest in hormonal variations, genetic predispositions, and disease presentations.^39^^,^^40^ Socially, gender roles and norms shape health behaviors and access to care. Men may underutilize preventive services due to cultural expectations of stoicism,^41^^,^^42^ while women may face barriers in accessing and affording healthcare resources.^43–45^ These intertwined factors highlight the necessity of accounting for gender in future student-led health promotion initiatives to ensure referrals to appropriate healthcare services are both effective and equitable.

### What is already known on this topic

In this study, age was found to be negatively correlated with follow-up, with younger participants being more likely to follow up after receiving ‘Pit Stop for Health’ services. This finding is supported by additional Australian statistics that details one in ten people aged 65 and over (11%), compared to one in four people aged 15–24 (27%) were more likely to visit a medical specialist.^46^ This finding may be explained by older people valuing relationships more than health, with past research confirming a strong link between social support, emotional and physical wellbeing.^47–49^ Even though our findings suggest younger participants were more likely to follow up, a systematic review (*n = 19*) reports younger people (*<*21 years) were frequently reported as more likely to miss appointments with their general practitioner.^50^ However, they are more likely to take up spontaneous health interventions, such as ‘Down to test’ a STI prevention program that was held at outdoor music festivals in New South Wales (Australia) between 2017 and 2020.^51^ Of note is the outlier in Table 2 reporting undertaking 11 health checks per year. This may be attributed to some participants including specialist consultations, urgent care visits, or follow-up appointments as separate health checkups, leading to inflated counts. In rare cases, individuals managing chronic conditions or undergoing ongoing treatment may have legitimately attended multiple distinct checkups and interpreted each as the ‘number of health check per year.’

### What this study adds

The results of this study suggest that while there is a strong and statistically significant effect on future follow-up and health modification behaviors, self-reported levels of health importance does not show a significant relationship with health modification. This discrepancy may be explained by bias and influential external factors. Self-reported measures are often subject to biases such as social desirability or inaccurate self-assessment, which may not accurately reflect actual behaviors.^52^ Also, the motivation to modify health behaviors may be influenced more by external factors, such as medical advice or immediate health concerns, rather than an individual’s perceived importance of health.^53^ It is possible that while individuals recognize the importance of health, translating this recognition into actionable health modifications requires additional support, resources, or interventions that were not accounted for in the self-reported measure.^54^ This highlights the complexity of health behavior change and the need for multifaceted approaches to effectively promote health modifications within reciprocal service-learning, student-led health promotion activities.

### Limitations of this study

This study has some limitations. Culture and ethnicity were not explored. Grouping of variables for the structural equation model assumed one-dimensionality, acknowledging the possibility that this grouping could introduce subject bias in data patterns and affect the determination of dimensionality. Normal consistency metrics are also a limitation. Construct validity might also be impacted if the grouped questions failed to adequately reflect the constructs they were intended to measure, potentially obscuring subtle differences when aggregated. To address these limitations, internal consistency was assessed alongside modeling, ensuring an excellent model fit to provide robust and reliable estimations based on the large sample size. Self-report bias, cross-sectional design, lack of generalizability, and absence of behavioral follow-up are also limitations of this study. However, given the model remains exploratory, caution in interpretation is warranted until further validation of constructs, paths, and relationships occur.

## Conclusion

The Pit Stop for Health initiative provides promising evidence that student-led, service-learning models can positively influence health behavior change at MGEs. Satisfaction with the service was a strong predictor of both behavior modification and the likelihood of recommending the initiative, aligning with constructs from the Theory of Planned Behavior and HBM. However, self-reported health importance did not correlate with behavior change, suggesting that external motivators may play a more significant role than internal perceptions. Age differences in follow-up behaviors, with younger participants more likely to engage, highlight the need for age-tailored health promotion strategies. The absence of gender effects, despite known biological and social influences, underscores the importance of further intersectional research. While these findings are encouraging, longitudinal studies or randomized controlled trials are needed to confirm the sustained impact and generalizability of student-led health promotion initiatives.

## Supplementary Material

fdaf159_revised_Supplementary_Appendix_2

## Data Availability

The data underlying this article will be shared on reasonable request to the corresponding author.
