# Supplementary material for: Positive effects of student-led health promotion activities at mass gathering events on health modification behaviors
Source: J Public Health (Oxf). 2025 Dec 23;48(1):347–57. doi: 10.1093/pubmed/fdaf159 (PMC13017314; doi:10.1093/pubmed/fdaf159)
Supplement: fdaf159_revised_Supplementary_Appendix_2 [file fdaf159_revised_supplementary_appendix_2.docx]

# Pit Stop for Health Questionnaire

## Demographic Information

1. Age Group

☐ 18–25  ☐ 26–35  ☐ 36–45  ☐ 46–55  ☐ 56–65  ☐ 66–75  ☐ 76–85  ☐ 85+

2. Gender Identity

☐ Male  ☐ Female  ☐ Non-binary  ☐ Transgender  ☐ Cisgender

☐ Other (please specify): ___________________________

3. Occupation Type

☐ Trade  ☐ Office Work  ☐ Hospitality  ☐ Caring for Others  ☐ Retail

☐ Other (please specify): ___________________________

## Health Perceptions and Behaviours

4. How important is your health to you?

☐ Extremely unimportant  ☐ Somewhat unimportant  ☐ Neutral  ☐ Somewhat important  ☐ Extremely important

5. How many times do you typically have your health checked in a year?

☐ 1  ☐ 2  ☐ 3  ☐ 4  ☐ 5  ☐ 6  ☐ 7  ☐ 8  ☐ 9  ☐ 10+

6. Were the results of your Pit Stop Health Check surprising to you?

☐ Yes  ☐ No  ☐ Other (please specify): ___________________________

7. Which areas of the health check did you score below the recommended level?

☐ Sleep  ☐ Alcohol Consumption  ☐ Blood Pressure  ☐ Exercise  ☐ Smoking

☐ Waist Circumference  ☐ Support Network  ☐ Testicle/Breast Screening

☐ Other (please specify): ___________________________

8. Would you consider modifying your health behaviours based on the Pit Stop results?

☐ Extremely unlikely  ☐ Somewhat unlikely  ☐ Neutral  ☐ Somewhat likely  ☐ Extremely likely

9. Are you likely to follow up with a doctor or health professional after visiting the Pit Stop?

☐ Extremely unlikely  ☐ Somewhat unlikely  ☐ Neutral  ☐ Somewhat likely  ☐ Extremely likely

10. Would you consider having an annual health check following your Pit Stop visit?

☐ Extremely unlikely  ☐ Somewhat unlikely  ☐ Neutral  ☐ Somewhat likely  ☐ Extremely likely

11. Are you likely to seek any other form of medical advice or assistance?

☐ Extremely unlikely  ☐ Somewhat unlikely  ☐ Neutral  ☐ Somewhat likely  ☐ Extremely likely

12. If yes, which area would this relate to?

☐ Sleep  ☐ Alcohol Consumption  ☐ Blood Pressure  ☐ Exercise  ☐ Smoking

☐ Waist Circumference  ☐ Support Network  ☐ Testicle/Breast Screening

☐ Other (please specify): ___________________________

## Feedback and Future Engagement

13. Would you visit the Pit Stop again?

☐ Extremely unlikely  ☐ Somewhat unlikely  ☐ Neutral  ☐ Somewhat likely  ☐ Extremely likely

14. Would you recommend the Pit Stop to friends or family?

☐ Extremely unlikely  ☐ Somewhat unlikely  ☐ Neutral  ☐ Somewhat likely  ☐ Extremely likely
